# Action Potential Initiation in the Hodgkin-Huxley Model

**DOI:** 10.1371/journal.pcbi.1000265

**Published:** 2009-01-16

**Authors:** Lucy J. Colwell, Michael P. Brenner

**Affiliations:** School of Engineering and Applied Science, Harvard University, Cambridge, Massachusetts, United States of America; University College London, United Kingdom

## Abstract

A recent paper of B. Naundorf *et al.* described an intriguing negative correlation between variability of the onset potential at which an action potential occurs (the onset span) and the rapidity of action potential initiation (the onset rapidity). This correlation was demonstrated in numerical simulations of the Hodgkin-Huxley model. Due to this antagonism, it is argued that Hodgkin-Huxley-type models are unable to explain action potential initiation observed in cortical neurons *in vivo* or *in vitro*. Here we apply a method from theoretical physics to derive an analytical characterization of this problem. We analytically compute the probability distribution of onset potentials and analytically derive the inverse relationship between onset span and onset rapidity. We find that the relationship between onset span and onset rapidity depends on the level of synaptic background activity. Hence we are able to elucidate the regions of parameter space for which the Hodgkin-Huxley model is able to accurately describe the behavior of this system.

## Author Summary

In 1952, Hodgkin and Huxley described the underlying mechanism for the firing of action potentials through which information is propagated in the nervous system. Hodgkin and Huxley's model relies on the opening and closing of channels, selectively allowing ions to move across the membrane. In the original picture, the channels open independently of one another. A recent paper argues that this model is incapable of modeling a set of action potential data recorded in the cortical neurons of cats. Instead the authors suggest that to model their data it is necessary to conclude that ion channels open cooperatively, so that opening one channel increases the chance that another channel opens. We analyze the initiation of action potentials using a method from theoretical physics, the path integral. We demonstrate that deviations of the data from the predictions of the Hodgkin-Huxley model hinge on measurement of the noise strength.

## Introduction

In 1952, Hodgkin and Huxley explained how action potentials are generated through the electrical excitability of neuronal membranes [1]. Action potentials arise from the synergistic action of sodium channels and potassium channels, each of which opens and closes in a voltage dependent fashion. A key feature of their model is that the channels open independently of each other; the probability that a channel is open depends only on the membrane voltage history.

A recent paper [2] challenged this picture. Therein the dynamics of action potential initiation in cortical neurons *in vivo* and *in vitro* are analyzed. The authors focus on two variables, the onset potential, i.e. the membrane potential at which an action potential fires, and the onset rapidity, or rate with which the action potential initially fires. Naundorf *et al.* argue that the variability or span of onset potentials observed in experiments, in conjunction with their swift onset rapidity, cannot be explained by the Hodgkin-Huxley model. In particular, within the Hodgkin-Huxley model they demonstrate through numerical simulations an antagonistic relationship between these two variables. If parameters are adjusted to fit the onset rapidity of the data, the observed onset span disagrees with the model, and vice versa. To fix this discrepancy [2] argues for a radical rethinking of the basic underpinnings of the Hodgkin and Huxley model, in which the probability of an ion channel being open depends not only on the membrane potential but also on the local density of channels.

The result reported in [2] was critically analyzed in a recent letter of D. A. McCormick *et al.* [3]. In [3] it was proposed that the observed combination of large onset span and swift onset rapidity could be captured using a Hodgkin-Huxley model if action potentials were initiated at one place within the cell, (the axon initial segment), and then propagated around 30 microns to the site at which they were recorded, (the soma). Whole-cell recordings from the soma of cortical pyramidal cells *in vitro* demonstrated faster onset rapidity and larger onset span then those obtained from the axon initial segment. This seemingly compelling reappraisal of the original data was in turn dissected by Naundorf *et al.* in [4] where it is suggested that the physiological setting of [3] is unrealistic, and the model inadequate.

Here we use a standard technique from theoretical physics (the path integral) to derive an analytical formula relating the onset rapidity and onset span. Our analysis applies to the classical Hodgkin-Huxley model, in addition to generalizations thereof, including those in which the channel opening probability depends on channel density [2]. To derive an analytical characterization of this relationship, we directly compute the probability distribution of the onset potential and demonstrate how it depends on model parameters. The formula that we arrive at can be used to compare experimental observations with the parameter values incorporated into such models. As anticipated by [2], a broad class of ion channel models displays an inverse relationship between onset rapidity and onset span. We find that the parameter relating onset rapidity to onset span depends on the amount of synaptic background activity included in the model. Indeed, a range of background activity exists where the classical Hodgkin-Huxley model agrees with the experimental data reported in [2].

## Model

We first review the essential framework of Hodgkin-Huxley type models for action potential generation. The dynamics of the membrane potential 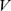 of a section of neuron, assumed to be spatially homogeneous, are given by [1]:
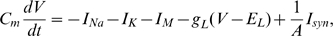
(1)where
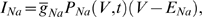


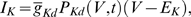


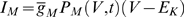
Here 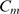 is the membrane capacitance, 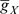 is the maximal conductance of channels of type 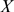, 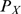 is the probability that a channel of type 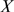 is open, 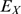 is the reversal potential for channel type 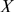 and the subscripts 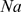, 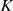 and 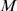 refer to sodium, potassium and M-type potassium channels respectively. A leak current is included with conductance 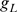 and reversal potential 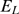, 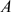 is the membrane area, while 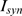 is the current resulting from synaptic background activity [5]. Background activity is typically modeled by assuming synaptic conductances are stochastic and consists of an excitatory conductance 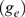 with reversal potential 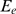 and an inhibitory conductance 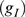 with reversal potential 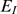, as found in [6] so that
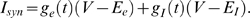
(2)In [2] the conductances 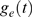 and 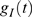 are modeled by Ornstein-Uhlenbeck processes with correlation times 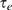 and 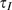, and noise diffusion coefficients 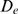 and 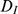 respectively [7].

We are interested in understanding from this model the relationship between onset span and onset rapidity, as defined by [2]. As described above, the onset rapidity is the rate at which the voltage increases; near onset the increase in voltage is exponential and so is given by the slope of a plot of 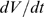 versus 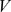. The onset span measures the variability of the voltage threshold for action potential initiation, [2] defines this threshold as the voltage at which 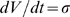, and takes 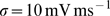. Due to the stochastic synaptic background, there is a distribution of voltages at which the voltage threshold is attained; the *onset span* is given by the width of this distribution. We calculate the probability distribution of voltage thresholds, and derive the onset span from the moments of this distribution.

## Results/Discussion

To proceed we use the fact that, at action potential initiation, we need only consider the sodium channels. This is because the potassium channels respond too slowly for their dynamics to influence the voltage 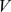 [8]. Moreover, near threshold, the probability that a sodium channel is open depends only on the membrane voltage 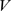. This probability is traditionally measured by the so-called activation curve [9], where 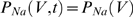. Under these assumptions, Eq. (1) reduces to
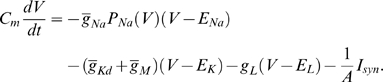
(3)Action potential onset occurs when 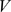 reaches 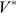, where 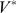 is an unstable equilibrium of Eq.(3) in the absence of noise. Below 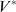 the membrane potential relaxes to its resting potential, whereas above 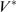 an action potential fires. To study the dynamics near onset, we therefore write 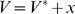, and expand equation (3) to leading order in 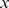, obtaining
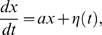
(4)where
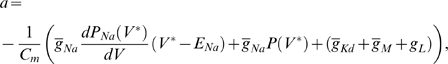
and
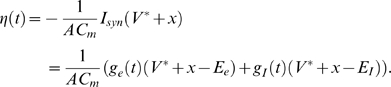
We use the parameter values 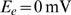 and 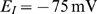 as found in [6] and used in [2]. Thus 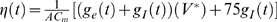. Near threshold the synaptic background itself is a single gaussian noise source with diffusion constant characterized by
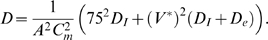
Note that in equation (4), 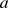 is the onset rapidity. According to [2],the voltage threshold is defined as the voltage at which 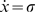, where 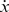 denotes the time derivative of 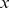. Owing to the noise source 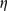 there is a range of 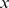 values at which this condition is attained. The onset span describes the range observed, and is related to the standard deviation of the probability distribution for these voltage thresholds.

Consider trajectories 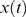 subject to the boundary conditions 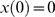 and 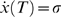, where 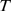 is the time at which the voltage threshold is attained. There is a distribution of times 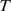 at which the threshold condition can be met. Moreover, for a given 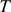, there is a distribution of voltages 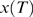 that the trajectory might attain at time 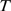. This distribution is characterized by a mean 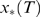, as well as a variance 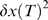. The total variance of the voltage threshold is therefore given by
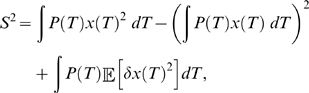
(5)where 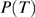 is the probability that the voltage threshold occurs at time 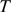, and 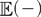 denotes the expectation. The first two terms of equation (5) make up the variance of mean values 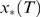 that occur owing to the range of times 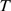 at which the threshold condition is met. For each such time 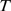, the final term sums the variance of voltages 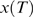 likely to be reached about the mean value 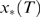.

Equation (5) is the fundamental equation for the onset span: it requires us to compute 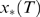, 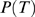 and 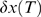. To proceed, we use the fact that the noise source 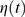 is Gaussian with variance 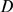, and therefore the probability density 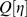 of a given realization 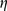 of the noise between 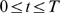 is
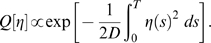
This leads to a path integral formulation of the probability of realizing a particular trajectory 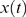 with 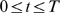, as developed in [10]. As equation (4) implies 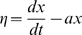, we find
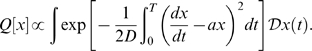
(6)


Here the integral is taken over all the possible paths that 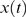 might take between time 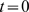 and 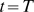. Some paths are of course more likely then others; application of the Euler-Lagrange equation finds that the most probable trajectory 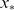 of Eq. (6) is the saddle point. It minimizes
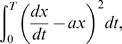
subject to the boundary conditions 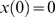 and 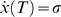 and therefore satisfies
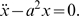
The most probable trajectory is the minimum of this quantity by definition. Since the probability density is of the form 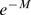, where 
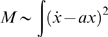
 is positive definite, the trajectory that minimizes 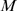 maximizes the probability. Imposing the boundary conditions we have
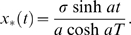
(7)We can use insert this solution into Eq. (6), in order to compute the probability density of this trajectory occurring. We obtain
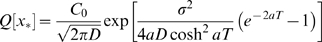
(8)


It is convenient to rewrite this formula by defining the dimensionless parameters 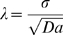 and 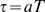. Since 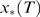 is a monotonic function of 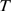 and thus also of 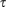 we can transform this to the probability density that the voltage threshold is achieved at time 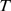, namely
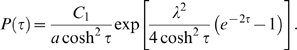
(9)In Eqs. (8) and (9) the constants 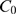 and 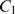 are set by the normalization condition.

We have now computed two of the three quantities needed to evaluate Eq. (5) for the onset span 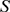. Thus we are able to evaluate the first two terms of this equations. Our theory has captured the probability distribution of the mean, but we also need to compute the variance about this mean in order to fully evaluate Eq. (5) for 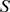. We can calculate this variance by noting that a general solution that satisfies 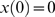 and 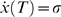 can be written as 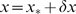, where 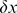 can be expanded in the Fourier series
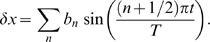



Substituting this into Eq. (6), we obtain
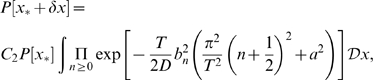
(10)where 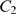 is a normalization constant. This demonstrates that the total probability distribution is a product of the probability for the mean trajectory 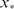, with Gaussian probability distributions for each of the 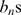. Now, Eq. (10) shows that each 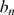 has mean zero and variance
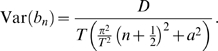
Hence the variance of 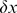 is given by
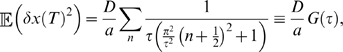
(11)where we have again used the dimensionless parameters 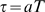 and 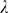 as defined above.

**Figure 1:**
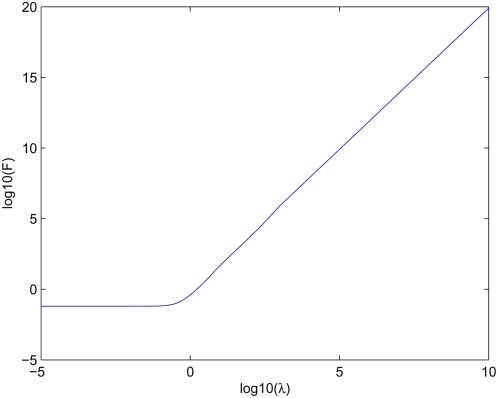
Evaluation of 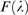. Numerical evaluation of the function 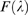 as defined in (13) above.

We now can evaluate Eq. 5 for 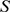. Taking Eqs. (7),(9) and (11) and letting 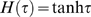 we have
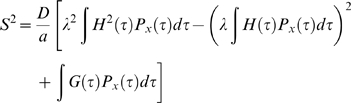
(12)

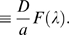
(13)The first two terms of Eq. (12) are the variance of the voltages reached by the mean path 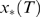, for each time 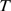 at which the threshold might be reached. The last term adds in the variance about the mean path for each value of 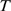, that is the variability from 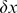. Equation (12) is the central result of this paper, directly relating the onset span 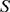 to the noise strength 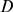, the voltage threshold 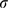 and the onset rapidity 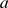. Figure 1 shows a numerical evaluation of 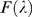.

Asymptotic analysis of the integral in Eq. (12) shows that at small 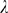, 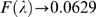, and at large 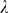, 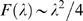 (Figure 1). Hence we obtain
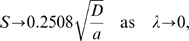
(14)

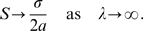
(15)We note that the low 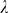 limit describes the behavior of a simple random walk; here a small value of 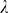 corresponds to a low threshold for the derivative. Thus the variance of onset voltages is simply the variance of all possible trajectories the random walk might take. In the high 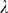 limit the size of the noise term ceases to much affect the variance of onset voltages. As the derivative threshold is high in this case, the deterministic exponential growth behavior will dominate those trajectories that reach the threshold.

**Figure 2:**
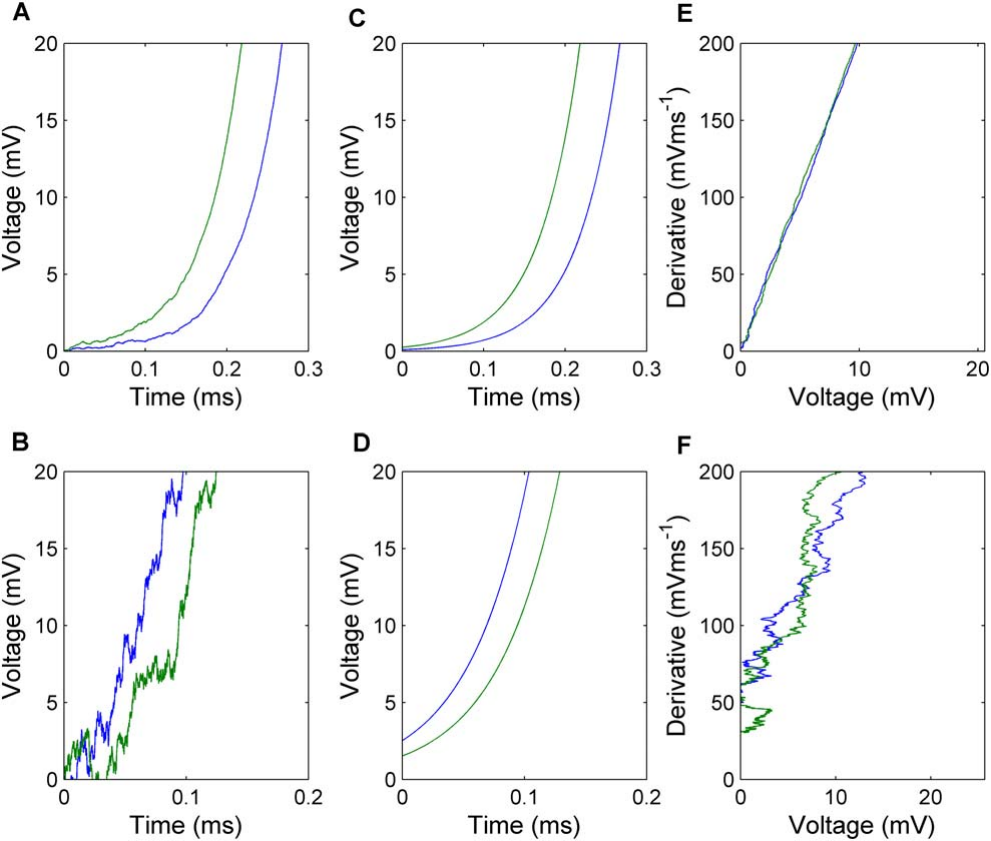
Simulation and differentiation of trajectories with different noise strengths. Pairs of trajectories simulated using (4) with (A) onset rapidity 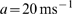 and noise 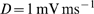 and (B) 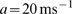 and 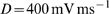. (C,D) As described in the text, an exponential curve was fit to each of the trajectories simulated. (E,F) The calculated derivative of the trajectories in A) and B) plotted as function of the voltage.

Thus we have calculated the variance of voltages at which action potential onset occurs as a function of the onset rapidity 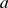, the onset threshold 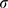 and the level 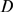 of synaptic background activity present. In Figure 2A we have simulated a pair of trajectories with parameter values 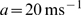 and 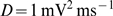, and in Figure 2B a pair with parameter values 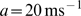 and 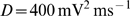. To ascertain the onset potential of each simulated trajectory we need to find the voltage at which the derivative of the trajectory first exceeds the threshold. As the model in (4) is not differentiable, it is necessary to fit a ‘smoothed’ curve to each trajectory, and find the derivative of this curve. In Figure 2C and 2D we have fitted an exponential curve with equation 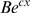 to each simulated trajectory. Figure 2E and 2F show the derivative extracted as a function of the voltage.

**Figure 3:**
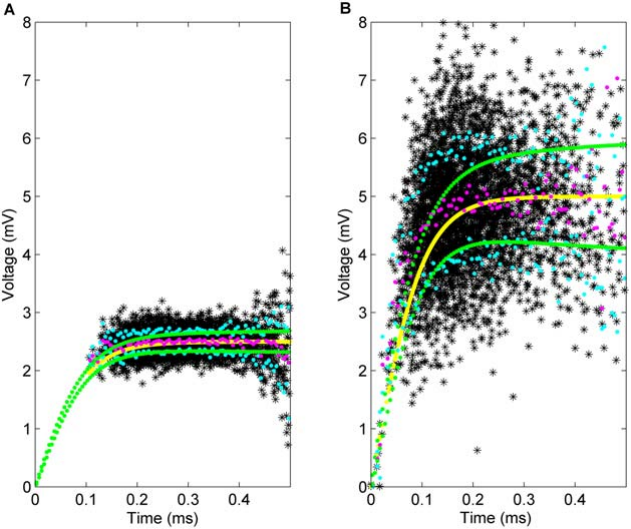
Comparison of simulations and theory for different parameter values. Trajectories (10000) were simulated as in Figure 2 with the following sets of parameter values: (A) 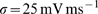, 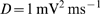 and 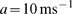, (B) 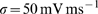, 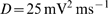 and 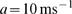. On each plot the endpoints were grouped into bins of width 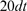. The average voltage in each bin is plotted in magenta, this should be compared with the most likely onset voltage at each time point according to our analysis, plotted in red. Similarly the standard deviation in each bin is plotted in cyan, and can be compared with the standard deviation predicted by our analysis at each time point, plotted in green.

To demonstrate the validity of our analysis, we use the reduced Hodgkin Huxley model described by (4) to simulate trajectories and compare the onset span we observe for particular sets of parameter values with that predicted by our analysis. In order to simulate the gaussian noise source 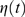 in Eq. (4) we use a Wiener process with the appropriate diffusion constant. In Figure 3 we choose two sets of parameter values and compare the range of onset potentials found by simulation with that predicted by our analysis. The black stars are the points at which each trajectory crossed the derivative threshold. On each plot the endpoints were grouped into bins of width 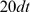. The average voltage in each bin is plotted in magenta, while the mean onset voltage at the center of each bin as predicted by our analysis is plotted in red. Similarly the standard deviation about the mean in each bin is plotted in cyan, and can be compared with the standard deviation predicted by our analysis which has been plotted in green. We observe that both the mean onset potential and the standard deviation about the mean at each time point found in the simulations is well matched by that predicted by our analysis.

In both the low 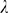 limit and the high 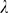 limit we found in Eq. (14) that there is indeed an antagonistic relationship between 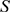 and 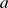, as argued by Naundorf *et al.* [2]. They observed that changing the parameters of the activation curve and the peak sodium conductance led to antagonistic changes in the onset rapidity and the onset span; hence they were not able to fit the Hodgkin-Huxley model to their data. Equations (14) and (15) show that the antagonistic relationship between 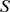 and 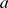 is controlled by 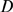 in the limit of low 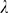, and 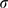 in the limit of high 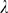. Neither 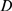 (the variance of the synaptic noise strength) nor 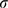 (the criterion for the voltage threshold) were varied in the simulations of Naundorf *et al.* [2]. We observe that our analysis can also be applied to the cooperative model proposed in [2], in which the probability of channel opening depends on both the membrane voltage, and the local channel density. In the vicinity of the unstable fixed point, incorporating the local channel density alters the value of 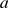, but does not change the form of equation (4).

We now compare the theory to the results of Naundorf. In their experiments, they measure the onset span as the difference between the maximum and minimum voltage threshold that is measured. Since 99.7% of observations fall within three standard deviations of the mean, we can approximate the onset span of between 50 and 500 trials as six times the standard deviation 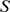. We assume that the calculation of the onset span from the simulations in Naundorf was done in the same fashion.

**Figure 4:**
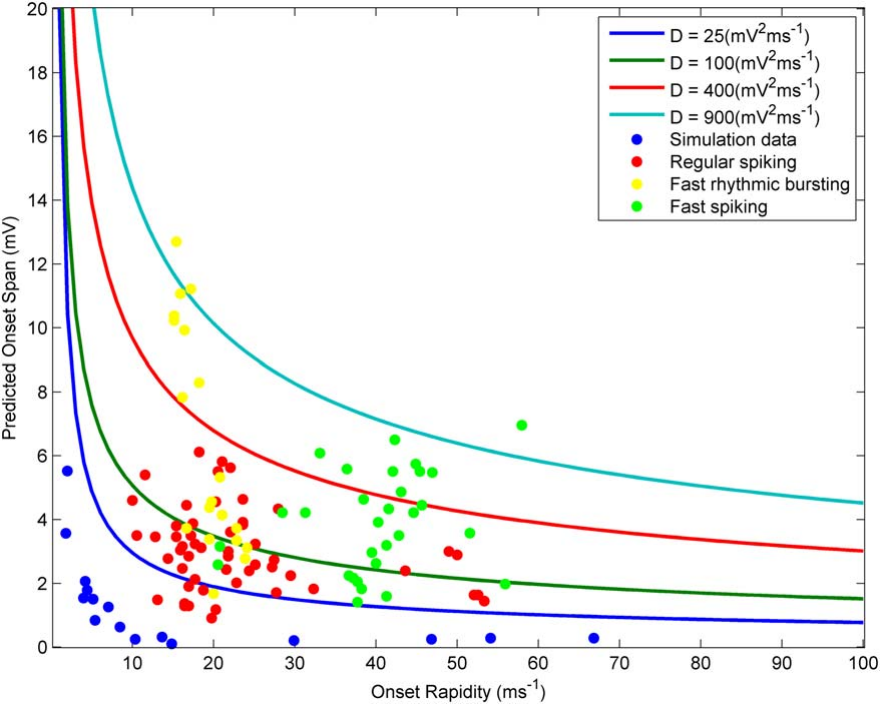
The relationship between onset span and onset rapidity as derived in the text. Here the solid blue dots are the simulation data points reported in [2], while the solid red, yellow and green dots are the experimental data points from [2] for cat visual cortex neurons classified electrophysiologically as regular spiking, fast rhythmic bursting, and fast spiking respectively. Data from many cells of each type is displayed in this plot. The curves show our analytical results for various values of the parameter 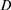.

In Figure 4 we have calculated the onset span as a function of 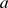 using different values of 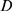. Changing the noise strength allows the theoretical curves to move between the various regimes observed experimentally. For most of the curves through the experimental data, a noise diffusion constant of around 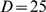 to 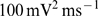 fits the data well. Although this is a larger diffusion constant than that apparently used in the simulations of Naundorf *et al.*, this value does a good job of emulating the experimental trajectories shown in Figure 2C and 2D of [2]. Figure 2B shows a simulated trajectory with noise strength 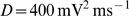 while Figure 2A shows a simulation with a smaller diffusion coefficient of 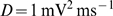. The voltage trace at 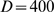 is visually similar to the behavior in Figure 2B of Naundorf in the vicinity of the unstable fixed point, whereas Figure 2A does not compare well, the noise level is much too low. Note that because we have linearized around the unstable fixed point, we can only expect to capture the behavior around the voltage threshold; this is presumably the reason that our simulations in Figure 2 do not reproduce the vertical spiking behavior occurring after action potential onset in Figure 2B of [2].

It is worth noting that additional sources of variance exist when comparing the experiments to the theory. In particular, (i) the theory assumes that the voltage threshold occurs precisely when 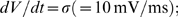 in contrast the experimental data show substantial variability in 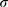. Additionally (ii) experiments report an *averaged* onset rapidity, whereas our analysis indicates a direct relationship between the onset potential and 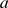. Both factors (i) and (ii) artificially increase the onset span.

The calculations described here clarify that to understand whether the experimental data is consistent with the Hodgkin Huxley picture, it is necessary to understand the corresponding level of 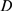; ideally, independent measurements of the synaptic background statistics are required. Intense levels of background activity characterized by high amplitude membrane potential fluctuations are known to occur during active states in neocortical neurons [11]. Combining the theoretical formalism described herein with measurements of the variance of synaptic conductances [12], while carefully controlling for other sources of variability in the measurement, is an excellent direction for future research.
